# Oxidative stress markers in bipolar disorder and first-degree relatives: differential associations of ischemia-modified albumin and superoxide dismutase

**DOI:** 10.3389/fpsyt.2026.1885863

**Published:** 2026-06-26

**Authors:** Ece Buyuksandalyaci Tunc, Burcu Kok Kendirlioglu, Hidayet E. Arat-Çelik, Esma Corekli Kaymakci, Serhat Tunc, Suat Kucukgoncu, Salim Neselioglu, Ozcan Erel

**Affiliations:** 1 Department of Psychiatry, Faculty of Medicine, Maltepe University, Maltepe, Türkiye; 2 Department of Psychiatry, Faculty of Medicine, Yeditepe University, İstanbul, Türkiye; 3 Department of Clinical Biochemistry, Faculty of Medicine, Ankara Yıldırım Beyazıt University, Ankara, Türkiye

**Keywords:** bipolar disorder, first-degree relatives, ischemia-modified albumin, oxidative stress, superoxide dismutase, thiol, disulfides

## Abstract

**Background:**

Oxidative stress has been implicated in the pathophysiology of bipolar disorder (BD), potentially contributing to neuroprogression and cellular dysfunction. However, whether specific oxidative stress markers reflect disease expression or familial vulnerability remains unclear. This study aimed to evaluate thiol-disulfide homeostasis (TDH), ischemia-modified albumin (IMA), ferroxidase, and superoxide dismutase (SOD) levels in patients with BD, their unaffected first-degree relatives (FDRs), and healthy controls (HCs).

**Methods:**

In this cross-sectional study, 50 patients with BD, 40 unaffected FDRs, and 50 HCs were included. Psychiatric diagnoses were confirmed using SCID-5, and remission was defined by Young Mania Rating Scale and Hamilton Depression Rating Scale scores <7. Fasting blood samples were analyzed for TDH parameters, IMA, ferroxidase, and SOD. Group differences were assessed using appropriate statistical tests, and multivariable linear regression analyses were performed, adjusting for age, sex, smoking status, and body mass index. Additional models further adjusted for medication use. Receiver operating characteristic (ROC) analyses were conducted to evaluate discriminative performance.

**Results:**

IMA levels were significantly higher in BD patients than in HCs and remained significant after adjustment for potential confounders (β = 0.17, p = 0.007). No significant difference in IMA levels was observed between FDRs and HCs. SOD levels were significantly elevated in both BD (β = 0.37, p = 0.015) and FDR groups (β = 0.41, p = 0.009) compared with HCs. After additional adjustment for medication use, the difference remained significant only in FDRs. Initial differences in thiol parameters between BD and FDR groups were no longer significant after adjustment, with age emerging as a major determinant. Ferroxidase levels did not differ significantly between groups. ROC analyses demonstrated modest discriminative performance for IMA and SOD (AUC range: 0.63–0.66).

**Conclusions:**

Elevated IMA levels in BD may reflect disease-related oxidative stress, whereas increased SOD levels observed in both BD patients and unaffected FDRs raise the possibility that SOD may be associated with familial vulnerability. However, the modest discriminative performance of these biomarkers and the cross-sectional design warrant cautious interpretation. Longitudinal studies are needed to clarify their potential role as biomarkers of disease-related oxidative stress and familial risk in BD.

## Introduction

Bipolar disorder (BD) is a long-term psychiatric disorder marked by recurrent episodes of depression and mania (1). There is increasing evidence that the pathophysiology of BD involves oxidative stress, potentially leading to neuronal damage, mitochondrial dysfunction, and inflammation (2).

Reactive oxygen species (ROS) are produced during normal cellular metabolism. When ROS production overcomes antioxidant defense capabilities, oxidative stress develops. Lipids, cell membranes, proteins, and DNA can all be compromised by oxidative stress, leading to protein degradation, loss of function, and DNA base changes and strand breaks (3–6). Specifically, this mechanism damages brain tissue and has been linked to the emergence of neurodegenerative and psychiatric disorders like schizophrenia and BD (7, 8).

Antioxidant systems counteract the harmful effects of ROS and consist of enzymatic (SOD, CAT, GPx, GR, PRxs) and non-enzymatic systems. Increased antioxidant enzyme activity may be a compensatory reaction to oxidative stress, and these pathways are crucial for preserving cellular redox homeostasis (9, 10). Superoxide dismutase (SOD) is one of these essential enzymes in the antioxidant defense system. Previous studies have reported altered SOD activity in BD; notably, Kunz et al. observed elevated SOD levels in patients during manic and depressive episodes, but not during the euthymic phase (11).

Thiol-disulfide homeostasis (TDH) is a dynamic indicator of oxidative stress, in which natural thiol (SH) groups react with ROS to form disulfide (SS) bonds. Enzymes like glutaredoxin (Grx) and thioredoxin (Trx) maintain this equilibrium. Previous studies have reported altered TDH in BD, including lower native thiol (SH) and total thiol (SH+SS) levels and, in some reports, higher SS levels and altered thiol/disulfide ratios compared with HC. Studies including individuals with BD and their first-degree relatives (FDR) have also reported similar changes (12–17).

Albumin is the main circulating protein with binding and antioxidant properties; it binds metal ions via its N-terminal region. However, this structure is disrupted by oxidative stress and ischemia, which lowers its binding capacity and causes ischemia-modified albumin (IMA) to develop. Increased IMA levels have been suggested as an indicator of oxidative stress in various medical and psychiatric conditions (18, 19). Elevated IMA levels have been reported in mood and anxiety disorders; for example, Tunç et al. found increased IMA levels in patients with mood disorders (18), Karaaslan et al. reported elevated IMA levels in major depressive disorder (20), and Sahin et al. demonstrated increased IMA levels in generalized anxiety disorder and panic disorder (21). In BD specifically, Korkmaz et al. reported higher IMA levels during mania compared with HC, with levels remaining elevated even in early remission, suggesting that IMA may reflect persistent oxidative stress in BD (15).

An increasing amount of research indicates that oxidative stress, neuroinflammation, and impairments in antioxidant defense systems may be involved in the biochemical mechanisms underlying neuropsychiatric conditions such as major depression, schizophrenia, and BD (22). Because oxidative stress results from an imbalance between oxidants and antioxidants, measuring oxidative stress typically involves quantifying oxidants and antioxidants, either directly or indirectly (20). Urine can be used to quantify systemic oxidative stress as oxidative stress-induced nucleoside damage, specifically as 8-oxo-7,8-dihydro-2′-deoxyguanosine (8-oxodG), its tautomer 8-hydroxy-2′-deoxyguanosine (8-OH-dG), and 8-oxo-7,8-dihydroguanosine (8-oxoGuo). The idea that oxidative stress-induced nucleotide damage is a familial risk-related trait in BD is supported by studies showing that levels of these oxidative stress markers are higher in BD patients and unaffected FDR than in HC (6, 16, 23, 24).

The biomarkers selected in this study represent complementary components of oxidative stress and antioxidant defense pathways. TDH parameters provide a dynamic measure of systemic redox balance through the reversible conversion of thiol groups to disulfide bonds under oxidative conditions. IMA reflects oxidative modification of albumin and has been proposed as an indirect marker of oxidative stress in several medical and psychiatric conditions. SOD is a major enzymatic antioxidant defense mechanism involved in the detoxification of superoxide radicals, and altered SOD activity has been reported in BD, although findings vary across illness phases and clinical populations. Ferroxidase activity, largely related to ceruloplasmin, reflects antioxidant processes involved in iron metabolism and may provide additional information regarding oxidative and metabolic regulation. Therefore, evaluating these markers together in BD patients, unaffected FDR, and HC may provide a broader understanding of oxidative stress alterations and help determine whether these abnormalities are primarily associated with disease expression or are also observable in individuals with familial risk. We hypothesized that oxidative stress and inflammatory processes may alter the levels of TDH, IMA, ferroxidase, and SOD in BD. Additionally, we propose that FDR who are unaffected by BD exhibit increased oxidative stress compared to healthy controls (HC), reflecting a potential familial vulnerability. The aim of this study was to evaluate these biomarkers in BD patients, FDR, and HC and examine their association with disease risk. Targeting oxidative stress pathways may contribute to a better understanding of disease mechanisms and familial risk in BD.

## Materials and methods

In this cross-sectional study, 50 patients with BD type 1 in remission, 40 unaffected FDR consisting of 34 siblings and 6 parents/children, and 50 HCs were enrolled. Participants were selected from the Maltepe University Psychiatry Clinic’s inpatient and outpatient services. Remission was defined as a minimum of 8 weeks, with YMRS and HDRS scores below 7. The study included unaffected first-degree relatives (FDR), namely parents or siblings. The Maltepe University Faculty of Medicine’s Clinical Research Ethics Committee approved the study protocol (No: 2023/900/52; December 29, 2023).

### Inclusion/exclusion criteria

A diagnosis verified by SCID-5, being between the ages of 18 and 65, and giving written informed consent were requirements for inclusion in the BD group, being at least a primary school graduate, signing the informed consent form and not having any prior psychiatric diagnosis for HC and FDR.

The exclusion criteria were: alcohol and substance abuse in any group, any psychiatric disorder in the HC group and the FDR group, intellectual disability, severe neurological (history of head trauma, history of bleeding, neurodegenerative diseases- dementia, Multiple sclerosis, Parkinson, Myasthenia Gravis, etc.), and chronic diseases (diabetes mellitus, hypo-hyperthyroidism) in all groups. Eight patients from BD, ten individuals from FDR, and four individuals from HC were excluded from the study due to chronic illnesses (Diabetes mellitus, hyper-hypothyroidism). None of the participants were receiving anti-inflammatory medications or dietary supplements at the time of the study.

### Psychometric scales to be used in the psychiatric examination

#### Sociodemographic and clinical information form

Sociodemographic and clinical data were collected using a structured form developed by the research team, including sociodemographic characteristics, age at the onset of psychiatric treatment, number of hospitalizations, current neuroleptic treatment, family history of psychiatric disorders, and history of physical illness. To determine whether patients were in remission, the Young Mania Rating Scale (YMRS) (25, 26), and Hamilton Depression Rating Scale (27)(HDRS Akdemir) adapted into Turkish, were administered. The SCID-5 (Structured Clinical Interview for DSM-5) is a standardized diagnostic evaluation, and the Turkish version was performed (28, 29).

#### Procedure

Data were collected using a study-specific form that included sociodemographic and clinical characteristics of patients, FDR, and HC. Samples of fasting venous blood were drawn into EDTA tubes between 06:00 and 07:00 am. Following centrifugation, serum samples were separated and stored at -80 °C at the Department of Biochemistry, Maltepe University Faculty of Medicine, until analysis. The samples were subsequently transported under cold-chain conditions to the Department of Clinical Biochemistry, Faculty of Medicine, Ankara Yıldırım Beyazıt University, Ankara, Türkiye, where all biochemical analyses were performed.

#### Measurement of TDH parameters

Erel & Neselioglu’s automated spectrophotometric approach was used to measure TDH parameters. Briefly, sodium borohydride generated free functional thiol groups and reduced SS bonds. To stop 5,5’-dithiobis(2-nitrobenzoic) acid (DTNB) from being reduced, excess sodium borohydride was used up and transformed into formaldehyde. Both SH and reduced thiol groups were detected after the reaction with DTNB. The dynamic SS amount was determined by taking half of the difference between SH and SH+SS. After measuring SH and SH+SS, we calculated the SS levels, SS/SH+SS, SS/SH, and SH/SH+SS (3).

#### Measurement of IMA

IMA levels were measured using the albumin-cobalt binding test. In this test, 200 µL of serum was mixed with 50 µL of 0.1% cobalt (II) chloride (CoCl_2_·6H_2_O), and the mixture was then incubated to enable albumin-cobalt binding. Following a further incubation period, 50 µL of 1.5 mg/mL dithiothreitol and 1.0 mL of 0.9% sodium chloride solution were added. Dithiothreitol was substituted with distilled water for the blank sample. A spectrophotometer was used to detect absorbance at 470 nm, and the results were reported in absorbance units (ABSU) (30).

#### Measurement of SOD

The stock solutions were 2 mM pyrogallol diluted in 0.01 N HCl, 9.8 mM DETAPAC adjusted to pH 7.0 with sodium hydroxide, and 0.1 M Tris-cacodylate buffer (pH 8.5). Before being used, these solutions were thawed after being kept at -20 °C. Pyrogallol was added to a reagent mixture containing 8.3 mL of tris-cacodylate buffer and 1.7 mL of DETAPAC. Pyrogallol was added to the reaction mixture, which comprised 50 mM Tris-cacodylate buffer and 1 mM DETAPAC, to start the reaction and reach a final concentration of 0.2 mM. The volumes of the sample, diluent, and reagent were 25 µL, 30 µL, and 150 µL, respectively. SOD activity was evaluated at 420 nm following a 10-minute incubation period (9).

#### Measurement of ceruloplasmin/ferroxidase

Ferroxidase activity (ceruloplasmin) was measured using an automated colorimetric method. The enzymatic oxidation of ferrous ions to ferric ions is the basis of this automated, colorimetric technique. Units per liter of serum were used to express the results (31).

### Statistical method

When appropriate, descriptive statistics were presented as mean ± standard deviation or median (min-max). The Kolmogorov-Smirnov and Shapiro-Wilk tests were used to evaluate the variables’ distribution. ANOVA or Kruskal-Wallis tests were used to compare groups, and then the relevant *post hoc* analyses were carried out. Quantitative independent variables with non-normal distributions were examined using the Kruskal-Wallis test. Qualitative independent data was analyzed using the chi-square test. Bonferroni correction was used in the *post hoc* analysis to take multiple comparisons into consideration. SPSS 28.0 was used for the analyses. The units of SH, SH+SS and SS tests were µmol/L. SS/SH, SS/SH+SS and SH/SH+SS ratios are expressed as percentages (%), i.e., they have no units. The IMA unit is ABSU. Units of ferroxidase and SOD tests are U/L. Multivariable linear regression analyses were conducted to assess the independent effects of group differences on oxidative stress markers after adjustment for age, sex, smoking status, and BMI. In the primary model, each oxidative stress marker (IMA, ferroxidase, SOD, SH, SH+SS, and SS) was included as a dependent variable, while group (BD, FDR, HC) was entered as a categorical independent variable, with HC as the reference group. Model fit was evaluated using R², adjusted R², F statistics, and corresponding model p-values for each regression model.

To further assess the potential impact of pharmacological treatment, additional regression analyses were conducted by including medication variables (mood stabilizers, antipsychotics, and antidepressants) as covariates in the model, alongside age, sex, smoking status, and BMI.

The discriminative power of important biomarkers (IMA and SOD) was assessed using receiver operating characteristic (ROC) curve analysis. ROC analyses were conducted for both BD vs HC and FDR vs HC comparisons. To evaluate diagnostic performance, the area under the curve (AUC) was computed.

## Results

There were no significant differences in age or sex distribution among the BD, FDR, and HC groups (p> 0.05). Sociodemographic characteristics are summarized in **Table 1**. In the BD group, 64% of participants reported a family history of psychiatric disorders. The mean age at onset was 23.3 years. The mean illness duration was 165.84 months. The median total number of episodes was 5, including 2 manic and 2 depressive episodes. The median number of hospitalizations was 1, and suicide attempts were infrequent. Regarding current treatment, lithium was used by 48% of patients, 38% valproic acid, 64% antipsychotics, 18% lamotrigine, 4% carbamazepine, and 20% antidepressants. YMRS and HDRS scores were low, consistent with remission status.

**Table 1:** Sociodemographic information of participants.

Variable	Statistic	^1^ Bipolar Disorder	^2^ First Degree Relative	^3^ Healthy Control	p	Test
Age (years)	Mean ± SD	36.9 ± 8.4	35.7 ± 8.2	34.1 ± 7.7	0.246	A
	Median	36.5	34.5	33.0		
Gender	Female, n (%)	35 (70.0%)	25 (62.5%)	27 (54.0%)	0.256	X²
	Male, n (%)	15 (30.0%)	15 (37.5%)	23 (46.0%)		
BMI (kg/m²)	Mean ± SD	27.6 ± 5.0	27.4 ± 18.8	24.7 ± 3.0	**0.004**	K
	Median	26.5	24.5¹	24.9¹		
Marital Status	Married, n (%)	20 (40.0%)	25 (62.5%)	30 (60.0%)	0.055	X²
	Single, n (%)	27 (54.0%)	12 (30.0%)	20 (40.0%)		
	Divorced/Widowed, n (%)	3 (6.0%)	3 (7.5%)	0 (0.0%)		
Education (years)	Mean ± SD	14.3 ± 2.6	14.4 ± 3.1	14.5 ± 2.7	0.849	K
	Median	15.0	15.0	15.0		
Employment Status	Unemployed, n (%)	16 (32.0%)	7 (17.5%)	3 (6.0%)	**0.004**	X²
	Employed, n (%)	34³ (68.0%)	33 (82.5%)	47 (94.0%)		
Income	Low, n (%)	5 (10.0%)	2 (5.0%)	1 (2.0%)	**0.014**	X²
	Medium, n (%)	40 (80.0%)	24 (60.0%)	39 (78.0%)		
	High, n (%)	5² (10.0%)	14 (35.0%)	10 (20.0%)		
Smoking Status	Negative, n (%)	24 (48.0%)	28 (70.0%)	26 (52.0%)	0.091	X²
	Positive, n (%)	26 (52.0%)	12 (30.0%)	24 (48.0%)		
Cigarettes per Day	Mean ± SD	20.1 ± 10.3	12.3 ± 7.5	13.3 ± 6.6	**0.017**	K
	Median	20.0	10.0¹	14.5¹		
Family Psychiatric History	Negative, n (%)	16 (32.0%)	1 (2.5%)	41 (82.0%)	**0.001**	X²
	Positive, n (%)	34² (68.0%)	39 (97.5%)	9¹² (18.0%)		
YMRS	Mean ± SD	0.50 ± 1.02	0.05 ± 0.32	0.00 ± 0.00	**0.001**	K
	Median	0.00	0.00¹	0.00¹		
HDRS	Mean ± SD	1.00 ± 1.69	0.40 ± 0.96	0.00 ± 0.00	**0.001**	K
	Median	0.00	0.00¹	0.00¹²		

^A^ ANOVA / ^K^ Kruskal-wallis (Mann-whitney u test) / ^X²^ Chi-square Test.

¹ Significant difference from the Patient Group (p < 0.05), ² Significant difference from the Patient Relatives Group (p < 0.05), ³ Significant difference from the Control Group (p < 0.05).

Bold values indicate statistically significant differences (p < 0.05).

Thiol-related measures showed limited group differences: The BD group had lower levels of SH and SH+SS than the FDR group, whereas other thiol/disulfide parameters did not differ significantly across groups. SOD levels were higher in both the BD and FDR groups than in HC, while IMA levels were considerably higher in the BD group than in HC. Ferroxidase levels did not differ significantly among groups (**Table 2**).

**Table 2:** Oxidative stress parameters of participants.

Marker	Statistic	^1^Bipolar disorder	^2^First degree relative	^3^Healthy control	ANOVA p	Test	Comparison groups	Post hoc p-value
SH	Mean ± SD	320.92 ± 34.66	342.03 ± 32.79	329.46 ± 33.96	**0.013**	A	1 vs 2 1 vs 3 2 vs 3	0.01* 0.572 0.229
SH+SS	Mean ± SD	360.3 ± 39.51	383.65 ± 36.85	370.56 ± 38.1	**0.016**	A	1 vs 2 1 vs 3 2 vs 3	0.012* 0.492 0.306
SS	Mean ± SD	19.69 ± 3.22	20.81 ± 2.68	20.55 ± 2.7	0.138	A	1 vs 2 1 vs 3 2 vs 3	0.20 0.371 1.00
SS/SH	Mean ± SD	6.13 ± 0.72	6.08 ± 0.54	6.24 ± 0.57	0.435	A	1 vs 2 1 vs 3 2 vs 3	1.00 1.00 0.667
SS/SH+SS	Mean ± SD	5.45 ± 0.57	5.42 ± 0.42	5.54 ± 0.45	0.439	A	1 vs 2 1 vs 3 2 vs 3	1.00 1.00 0.687
SH/SH+SS	Mean ± SD	89.09 ± 1.14	89.16 ± 0.85	88.91 ± 0.91	0.431	A	1 vs 2 1 vs 3 2 vs 3	1.00 1.00 0.674
IMA	Mean ± SD	0.83 ± 0.35	0.76 ± 0.21	0.64 ± 0.3	**0.003**	A	1 vs 2 1 vs 3 2 vs 3	0.833 0.003* 0.131
Ferroxidase	Mean ± SD	1051.2 ± 307.2	1146.5 ± 228.3	1140.5 ± 233.1	0.143	K	1 vs 2 1 vs 3 2 vs 3	0.141 0.095 1.00
SOD	Mean ± SD	5.88 ± 0.76	5.94 ± 0.63	5.5 ± 0.73	**0.004**	A	1 vs 2 1 vs 3 2 vs 3	1.00 0.017* 0.011*

A, ANOVA/K Kruskal-Wallis (Mann-Whitney U test). ¹Significant difference from the Patient Group (p < 0.05), ²Significant difference from the Patient Relatives Group (p < 0.05), ³Significant difference from the Control Group (p < 0.05).

SH, Native thiol; SH+SS, Total thiol; SS, Disulfide; IMA, Ischemia-modified albumin; SOD, Superoxide dismutase.Bold values indicate statistically significant differences (p < 0.05).

* indicates statistically significant pairwise post-hoc comparison (p < 0.05).

To investigate these relationships further, multivariable linear regression analyses were performed, controlling for age, sex, smoking status, and BMI. IMA levels in BD patients were considerably higher than those in HC, even after controlling for confounders (β = 0.17, p = 0.007), whereas no significant difference was observed between FDR and HC. SOD levels remained significantly elevated in both BD (β = 0.37, p = 0.015) and FDR groups (β = 0.41, p = 0.009) compared to HC, indicating that this association was independent of covariates. No significant group differences in ferroxidase levels were found after adjustment; however, ferroxidase was significantly associated with age (β = -6.04, p = 0.024), sex (β = 162.21, p < 0.001), and BMI (β = -4.34, p = 0.032). For thiol parameters, the previously observed differences between BD and FDR groups were no longer significant after adjustment. Age was identified as the primary determinant of thiol levels, including NT (β = -1.33, p < 0.001), TT (β = -1.53, p < 0.001), and SS (β = -0.10, p = 0.001).

To further evaluate the potential effect of medications, additional analyses were performed, including medication classes (mood stabilizers, antipsychotics, and antidepressants) as covariates, in addition to age, sex, smoking status, and BMI. After this adjustment, IMA levels remained significantly higher in BD patients compared to HC (β = 0.19, p = 0.031). For SOD, there was no longer a significant difference between BD patients and HC (p = 0.262), whereas FDR continued to show significantly higher levels compared to HC (β = 0.39, p = 0.015). Ferroxidase and thiol parameters showed no significant group differences. However, ferroxidase levels were significantly associated with mood stabilizer use (β = 158.43, p = 0.032), while antipsychotic and antidepressant use showed no significant effects. However, after adjustment for age, sex, smoking status, and BMI, the differences in SH and SH+SS levels between the BD and FDR groups were no longer significant, and no significant associations were observed for SS levels, suggesting that these findings may have been influenced by confounding factors, particularly age (**Table 3**).

**Table 3:** Multiple linear regression analysis of oxidative stress markers.

Marker	Variable	β	95% CI lower	95% CI upper	p-value
IMA	BD vs HC	0.1702	0.0470	0.2935	**0.0072**
IMA	FDR vs HC	0.0976	-0.0319	0.2271	0.1383
IMA	Age	0.0016	-0.0049	0.0081	0.6238
IMA	Sex (male)	-0.0699	-0.1756	0.0358	0.1933
IMA	Smoking	-0.0212	-0.1271	0.0846	0.6919
IMA	BMI	0.0025	-0.0024	0.0074	0.3226
SOD	BD vs HC	0.3663	0.0731	0.6595	**0.0147**
SOD	FDR vs HC	0.4120	0.1040	0.7201	**0.0091**
SOD	Age	0.0003	-0.0151	0.0157	0.9667
SOD	Sex (male)	-0.1721	-0.4236	0.0794	0.1783
SOD	Smoking	0.0512	-0.2005	0.3030	0.6880
SOD	BMI	0.0031	-0.0086	0.0147	0.6048
Ferroxidase	BD vs HC	-33.3063	-133.2012	66.5886	0.5107
Ferroxidase	FDR vs HC	37.4393	-67.5301	142.4087	0.4817
Ferroxidase	Age	-6.0441	-11.2862	-0.8020	**0.0242**
Ferroxidase	Sex (male)	162.2070	76.4965	247.9179	**0.0003**
Ferroxidase	Smoking	-19.3212	-105.1038	66.4613	0.6567
Ferroxidase	BMI	-4.3436	-8.3118	-0.3754	**0.0322**
NT	BD vs HC	-2.4779	-15.7022	10.7464	0.7115
NT	FDR vs HC	13.2847	-0.6114	27.1808	0.0608
NT	Age	-1.3315	-2.0255	-0.6376	**0.0002**
NT	Sex (male)	10.0236	-1.3230	21.3702	0.0829
NT	Smoking	-4.3582	-15.7143	6.9979	0.4491
NT	BMI	-0.1317	-0.6570	0.3936	0.6207
TT	BD vs HC	-3.2641	-18.1181	11.5899	0.6645
TT	FDR vs HC	14.1935	-1.4151	29.8021	0.0743
TT	Age	-1.5325	-2.3120	-0.7530	**0.0002**
TT	Sex (male)	11.9065	-0.8384	24.6514	0.0668
TT	Smoking	-4.2528	-17.0083	8.5028	0.5107
TT	BMI	-0.1331	-0.7232	0.4569	0.6562
SS	BD vs HC	-0.3931	-1.5173	0.7311	0.4904
SS	FDR vs HC	0.4544	-0.7269	1.6357	0.4481
SS	Age	-0.1005	-0.1595	-0.0415	**0.0010**
SS	Sex (male)	0.9414	-0.0231	1.9060	0.0557
SS	Smoking	0.0527	-0.9127	1.0181	0.9142
SS	BMI	-0.0007	-0.0454	0.0440	0.9755

HC (healthy controls) was used as the reference group. All models were adjusted for age, sex, smoking status, and BMI. β values are unstandardized coefficients. CI, confidence interval; BMI, body mass index. Bold values indicate statistically significant differences (p < 0.05).

Additional regression analyses were performed within the BD group to evaluate the associations between oxidative stress markers and illness-related variables, including total number of episodes, remission duration, age at onset, and total illness duration, while adjusting for age, sex, smoking status, BMI, and medication use. No significant associations were identified between these clinical variables and the examined oxidative stress markers. Model fit statistics for the regression analyses are presented in **Table 4**. The models explained between 8.9% and 17.0% of the variance in oxidative stress markers.

**Table 4:** Model fit statistics for multiple linear regression models.

Marker	N	R²	Adjusted R²	F statistic	Model p-value
IMA	140	0.091	0.0501	2.2216	**0.0448**
SOD	140	0.089	0.0476	2.1574	0.0510
Ferroxidase	140	0.170	0.1323	4.5310	**0.0003**
NT	140	0.160	0.1221	4.2220	**0.0006**
TT	140	0.165	0.1271	4.3742	**0.0005**
SS	140	0.130	0.0908	3.3133	**0.0045**

R² represents the proportion of variance explained by the regression model, whereas adjusted R² accounts for the number of predictors included in the model. F-statistics and corresponding model p-values indicate the overall significance of each regression model. All models were adjusted for age, sex, smoking status, and BMI.

Bold values indicate statistically significant differences (p < 0.05).

**Figure 1:**
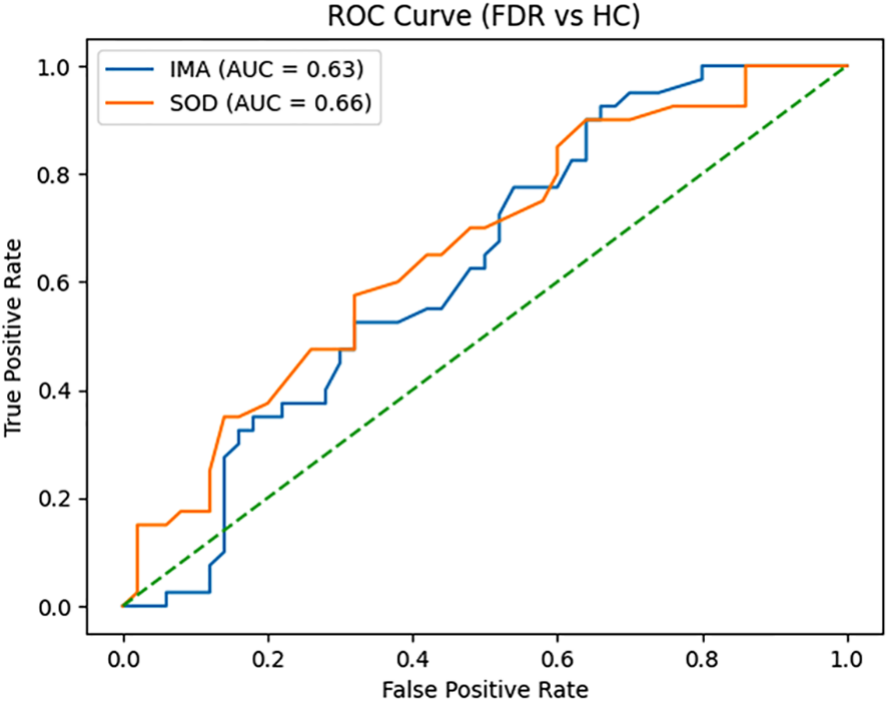
ROC curve for FDR vs HC.

**Figure 2:**
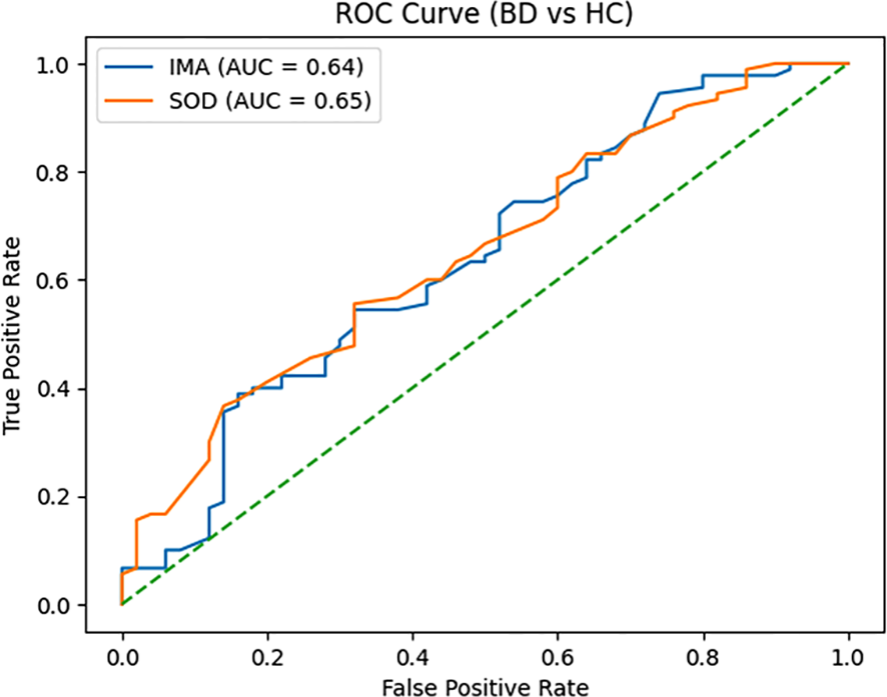
ROC curve for BD and HC.

Receiver operating characteristic (ROC) curve analysis was performed to evaluate the discriminative ability of IMA and SOD in distinguishing BD patients from HC. The area under the curve (AUC) was 0.64 for IMA and 0.65 for SOD, indicating a modest discriminative performance for both markers. ROC analyses were additionally conducted for FDR versus HC comparisons. The AUC was 0.63 for IMA and 0.66 for SOD, indicating limited discriminative ability (**Figures 1**, **2**).

## Discussion

In this study, we evaluated oxidative stress markers in patients with BD, FDR, and HC. Our results indicate that IMA levels are elevated in BD patients, consistent with disease-related alterations in oxidative stress. In contrast, the higher levels of SOD in both BD patients and the FDR group, and the persistence of this trend even after adjusting for sociodemographic and clinical variables, raise the possibility that SOD may represent a candidate trait-related marker associated with familial vulnerability; however, this interpretation should be considered cautiously given the cross-sectional design of the study. Furthermore, no significant associations were observed between oxidative stress markers and illness-related variables, including total number of episodes, remission duration, age at onset, and total illness duration. This finding may suggest that these biomarkers are not primarily driven by illness chronicity or cumulative episode burden in this sample; however, it should be interpreted cautiously given the cross-sectional design and limited sample size. ROC analyses showed limited discriminative power for IMA and SOD, suggesting that these markers may have limited clinical utility when used individually.

Prior studies have generally reported altered TDH in BD patients, characterized by lower SH and SH+SS levels and higher SS-related parameters compared to HC, and similar alterations have also been observed in FDR, suggesting that thiol-disulfide imbalance may be relevant to familial vulnerability (12, 16, 17). In contrast to previous reports, we did not find significant differences between BD patients and HC after adjustment, nor did we detect similar alterations in FDR. This difference may be attributable to differences in clinical state and methodological factors, as many previous studies included patients during acute mood episodes and did not fully account for potential confounders. Our findings, particularly the loss of significance after adjustment for age, suggest that thiol parameters may be sensitive to demographic influences and should therefore be interpreted cautiously in cross-sectional biomarker studies.

Our findings showed that IMA levels were higher in the BD group compared to the HC group, and this difference remained statistically significant after adjustment for age, sex, smoking status, and BMI, suggesting a potential association between IMA and disease-related oxidative stress in BD. IMA is recognized as a marker of ischemia and oxidative stress and has been reported to be elevated across psychiatric disorders, including major depressive disorder, schizophrenia and BD (18, 19). Previous studies have demonstrated that IMA levels remain elevated from acute mania into early remission, suggesting that oxidative stress may persist beyond symptomatic phases (15). However, in our study, no significant difference in IMA levels was observed between FDR and HC. This finding suggests that IMA may be more closely associated with disease-related oxidative stress than with familial vulnerability in this sample. While oxidative stress abnormalities have been consistently reported in FDR across various biomarkers (17), the lack of difference in IMA in our study suggests that not all oxidative markers may be equally informative regarding familial vulnerability. Overall, these findings support the possibility that IMA may be more closely related to disease-associated oxidative stress in BD, although longitudinal studies are needed to clarify whether IMA reflects state-related changes or more persistent oxidative alterations across illness phases.

Our study showed higher SOD levels in both BD patients and FDR compared to HC, which may represent a compensatory antioxidant response to increased oxidative stress. SOD plays a central role in detoxifying superoxide radicals, and its elevation may indicate a compensatory mechanism in response to oxidative imbalance. Notably, the persistence of elevated SOD levels in FDR, even after adjustment for sociodemographic and clinical variables, raises the possibility that this finding may be associated with familial vulnerability rather than disease status alone. However, when medication variables were included in the model, the difference between BD patients and HC was no longer significant, whereas the difference between FDR and HC remained significant, indicating that medication use may partially affect SOD levels in BD patients, whereas the elevation observed in FDR was not attributable to medication exposure in this sample. Evidence from meta-analyses suggests that SOD levels in BD are heterogeneous, with no significant overall difference compared to HC, and may vary according to illness phase, treatment status, and metabolic factors (10). This variability is consistent with our findings, in which the BD-HC difference was attenuated after adjustment for medication. Previous studies have also reported mixed results, with several studies demonstrating elevated SOD levels (10, 11) while others have reported lower levels, particularly in depressive episodes (32, 33). Additionally, SOD levels remained unchanged after ECT, indicating that certain treatments may not substantially affect this marker. These findings suggest that SOD and related oxidative stress markers may warrant further investigation as potential indicators of familial risk, particularly in individuals with a familial predisposition. Supporting this, evidence from studies on unaffected FDR consistently demonstrates increased oxidative stress burden, including elevated oxidative DNA and RNA damage, which persists over time and parallels findings in BD patients (23, 24, 34). The elevated SOD levels observed in relatives in our study, similar to those in the patient group, further raise the possibility that FDR may exhibit subclinical oxidative stress; however, longitudinal studies are needed to determine whether this reflects inherited vulnerability to oxidative imbalance.

These findings, although heterogeneous, suggest that oxidative stress pathways may remain relevant to the pathophysiology of BD and warrant further investigation as potential therapeutic targets. Furthermore, the elevated SOD levels observed in relatives, similar to those in the patient group, suggest that FDR may already exhibit subclinical oxidative stress, potentially reflecting an inherited vulnerability to oxidative imbalance. Unlike IMA and SOD, ferroxidase activity did not differ significantly between the groups. However, regression analyses revealed that ferroxidase levels were significantly associated with age, sex, BMI, and medication use, suggesting that this marker may be influenced by demographic and metabolic factors and may be less directly related to BD status in this sample. While ferroxidase is involved in iron metabolism and serves an antioxidant role, its lack of significant variation in this study contrasts with the findings of Tunç, who reported elevated ferroxidase activity in BD patients compared to those with major depressive disorder, schizophrenia and HC (35). This discrepancy may be attributable to differences in sample characteristics, including the inclusion of both BD type I and type II patients in previous studies, as well as differences in sample size. Additionally, Manto discussed how abnormalities in copper homeostasis, which influence ferroxidase activity, are related to neurodegeneration (36). This could suggest that ferroxidase abnormalities might be more relevant in neurodegenerative conditions or advanced stages of psychiatric disorders. In contrast, significant ferroxidase alterations may be less apparent in earlier phases of BD.

## Limitations

When evaluating these results, several limitations should be taken into account. First, the relatively modest and heterogeneous sample size represents an important limitation, particularly given the number of oxidative stress biomarkers and comparisons examined. Although significant group differences were detected for selected markers, no *a priori* sample size or power calculation was performed, as this study was designed as an exploratory biomarker investigation. Therefore, the sample size may have limited statistical power for some secondary and subgroup analyses, particularly medication-related effects. A larger and more diverse sample would provide more robust evidence and improve the generalizability of the findings to the broader BD population. Second, the study’s cross-sectional design makes it difficult to evaluate how oxidative stress markers change over time or across different stages of BD. Third, although we adjusted for several important covariates, including age, sex, smoking status, BMI, and medication use, residual confounding cannot be entirely excluded. In particular, the potential effects of different medication types, dosages, and treatment durations on oxidative stress markers could not be fully disentangled. In addition, lifestyle-related factors known to influence oxidative stress, including diet, physical activity, sleep characteristics, and alcohol consumption, were not systematically assessed. Although smoking status was included as a covariate, detailed measures of chronic smoking exposure that may contribute to oxidative stress, such as pack-year calculations, and nicotine dependence severity measures were not available. Finally, the discriminative ability of the examined biomarkers, as indicated by ROC analysis, was modest, suggesting limited clinical utility when used individually. Future longitudinal, multi-marker studies with larger and adequately powered samples are needed to establish the clinical utility of oxidative stress markers for BD diagnosis, progression, treatment monitoring, and risk assessment in FDR.

## Conclusion

In conclusion, our results indicate that oxidative stress markers, specifically IMA and SOD, may play distinct roles in BD. Elevated IMA levels in BD patients may reflect disease-related oxidative stress, whereas increased SOD levels observed in both patients and their FDR raise the possibility that SOD may be associated with familial vulnerability. In contrast, TDH parameters did not show consistent associations after adjustment, indicating that these findings may be largely influenced by demographic factors. These results may support a preliminary model in which IMA is more closely related to disease-associated oxidative stress, SOD may serve as a candidate marker of familial vulnerability, and thiol parameters may be more susceptible to demographic confounding. However, the modest discriminatory performance of IMA and SOD in ROC analyses indicates that these markers have limited clinical utility when used individually. Future longitudinal studies with larger samples and multi-marker approaches are needed to clarify the clinical relevance of these biomarkers for tracking disease progression and familial risk.

## Data Availability

The datasets presented in this article are not readily available due to patient privacy concerns. Requests to access the datasets should be directed to dr.ecetunc@gmail.com.
